# Macular, choroidal and disc associations across women’s reproductive life stages: a scoping review from menarche to post-menopause

**DOI:** 10.1038/s41433-025-03592-w

**Published:** 2025-01-15

**Authors:** Ana Paula Ribeiro Reis, Estelle Ioannidou, Kelsey V. Stuart, Siegfried K. Wagner, Paul J. Foster, Anthony P. Khawaja, Axel Petzold, Sobha Sivaprasad, Nikolas Pontikos, Pearse A. Keane, Konstantinos Balaskas, Praveen J. Patel

**Affiliations:** National Institute for Health Research Biomedical Research Centre at Moorfields Eye Hospital NHS Foundation Trust and UCL Institute of Ophthalmology, London, UK

**Keywords:** Retina, Epidemiology

## Abstract

Oestrogen and progesterone fluctuate cyclically in women throughout their adult lives. Although these hormones cross the blood-retinal barrier and bind to intraocular receptors, their effects remain unclear. We present the first review to date on associations between posterior pole structures—specifically the macula, choroid, and optic disc—and both the menstrual cycle and post-menopausal period, utilising multimodal imaging techniques in healthy adult non-pregnant women. We excluded studies on contraception and hormonal replacement therapy, focusing solely on physiological associations. Despite the comprehensive scope of our review, limited data and inconsistent reporting among studies prevented the establishment of meaningful trends. Across menstrual cycle phases, choroidal thickness (CHT) was the most consistently reported parameter, with thinning during the luteal phase compared to the follicular phase. Conversely, no significant differences were observed in macular or disc morphology across the cycle, likely reflecting a preserved structure despite potential fluctuations in blood flow and perfusion. Studies comparing pre- and post-menopausal associations, after adjusting for age or body mass index (BMI), failed to reveal meaningful trends, highlighting the difficulty in separating the effect of age from hormonal declines in older women. Understanding how hormonal cycles impact the posterior pole in women is crucial for addressing sex differences in various ocular pathologies. Research on female-specific factors is still sparse, and interestingly, the majority of affiliations in the reviewed articles did not originate from regions with the highest biomedical research funding and publication rates. We encourage further studies focusing on female-specific variables and provide recommendations for future designs.

## Introduction

Female sex steroid hormones, including oestrogen and progesterone, undergo cyclic changes in women from 13 [1] to 48–50 years of age [2], and are influenced by factors such as hormonal contraception, pregnancy, disease, medication, or extreme conditions. In the years preceding menopause, menstrual fluctuations become more irregular [3–5], until a permanent hormonal decline is established. Both oestrogen and progesterone cross the blood-retinal barrier [6] and have receptors in various parts of the eye, including the cornea, lens, iris, ciliary body, and retina, demonstrated in both rodents and humans [7, 8]. Oestrogen receptor alpha (ORα) was first identified in the neurosensory retina and retinal pigment epithelium (RPE) in post-mortem eyes of young women in 1999 [9]. Later, mRNA for ORα, oestrogen receptor beta (ORβ), and progesterone were discovered in the retina of both female and male subjects aged between 52 and 84 years, with variable expression levels [10, 11].

Sex-related variation in macular structure is apparent, yet still poorly understood. Women exhibit thinner retinas in both adults [12–15] and children [16], with differences in individual sublayers, particularly in the inner retina [15, 17–19]. Morphologically, they consistently present a broader and shallower foveal pit across different ethnicities compared to male counterparts [20–26]. While ovarian hormones predominate in women and are known to influence neuronal plasticity and growth [27–29], it remains unclear whether these retinal sex differences arise from true physiological variation or potential bias in retinal image scale estimation [25, 30] due to unaccounted sex disparities in cornea topography [31] and shorter axial length [32].

On the other hand, women also exhibit higher susceptibility or greater protection to some retinal diseases across different hormonal phases. Postmenopausal women are reported to have a higher incidence and faster progression of posterior vitreous detachment than men of the same age [33, 34] and idiopathic macular holes are more frequent in women, across different ethnicities [35–38]. Progestogenic hormones were shown to modulate pro-survival pathways in the retina in experimental acute brain injury and retinitis pigmentosa models [39–41]. Furthermore, in age-related macular degeneration (AMD), research suggested a beneficial role of oestrogen due to its antioxidant effects, with longer exposure to oestrogen or hormone replacement therapy (HRT) in post-menopause associated with a lower risk of developing late AMD [42–45]. Finally, female sex steroid hormones also seem to have a protective effect on the optic disc. In primary open-angle glaucoma, a longer reproductive period, with early menarche and/or late menopause, was associated with a reduced risk of disease [46–48] and conversely, early menopause was linked to a higher prevalence of glaucoma [49]. The prevailing hypothesis is that oestrogen may modulate aqueous humour dynamics and vascular tone through nitric oxide synthase, with relatively consistent associations between oestrogen-only HRT use and lower intraocular pressure [48].

In general, studies investigating ocular sex differences often demonstrate variability in study designs and reporting methods, with considerable risk of bias [48]. Understanding the physiological hormonal impact of fluctuating sex steroid hormones across women’s lifespans is a crucial first step in comprehending sex differences and standardising study methodologies. Menstrual cycle phases and menopausal status can be used as proxies for hormonal action [50, 51] and are frequently neglected. This is the first review to date to provide a comprehensive overview of associations between posterior pole structures—specifically the macula, choroid, and optic disc—and the menstrual cycle and post-menopausal period, utilising multimodal imaging techniques in healthy adult women.

## Methods

We conducted this scoping review to offer an overview and identify gaps in knowledge within the field of associations between menstrual and menopausal statuses and macular and disc parameters in healthy adult women. We followed the Preferred Reporting Items for Systematic Reviews and Meta-Analyses Extension for Scoping Reviews (PRISMA-ScR) statement [52] and their checklist adapted to this work is included in Supplementary Material 1. The protocol of this scoping review was registered in the Open Science Framework (OSF) platform on the 18th of February 2024 and is available at osf.io/bfwn3.

### Search methods for identifying studies

We developed a comprehensive electronic Boolean search string and applied it to two peer-reviewed biomedical databases: PubMed (www.ncbi.nlm.gov/pubmed) and Embase (https://ovidsp.dc1.ovid.com/). We constructed our search string by combining three concepts: first, macular and disc morphology; second, imaging modalities and related structural changes; and third, cycles influenced by female hormones. The complete search string for PubMed is provided in Supplementary Material 2, and a comparable search was applied in Embase. To maximise the scope, a forward citation search was conducted to identify further relevant papers, and keywords were extracted from grey literature repositories. The last search across all electronic databases was conducted on the 18th of February 2024.

### Eligibility criteria for considering studies for this review

We included studies that focused on healthy adult women, exploring differences and associations with retina and disc morphology across the menstrual cycle and menopausal-related factors. These studies assessed macular and/or disc morphology using current clinical multi-imaging modalities and could date back to the beginnings of the databases used. We excluded studies involving pregnancy, breastfeeding, hormonal contraception, HRT, treatments or supplementation of any kind. Additionally, we excluded studies that investigated cycle and menopause changes related to systemic or ocular disease or focused on differences between sexes rather than differences within women’s stages. However, if a study addressed any of these exclusions but also included a segment on healthy women, we included the study, considering only the relevant section. Experimental approaches, as well as functional or haemodynamic studies, were excluded.

### Study selection

All identified citations were downloaded from PubMed as*.nbib*, from Embase as*.ris* and a merged spreadsheet was then collated using Paperpile (© Paperpile LLC 2023). After eliminating duplicates, title and abstract screening underwent validation by two reviewers (A.P.R.R. and E.I.), with any discrepancies resolved through arbitration by a third reviewer (P.J.P). Initially, we excluded non-human or basic science studies, non-retrievable abstracts, and non-English publications. Subsequently, we thoroughly reviewed the remaining abstracts and, when needed, the full-text versions against our inclusion and exclusion criteria. Articles facing exclusion were categorised as off-topic and further divided into 9 subcategories: (1) contraception, (2) pregnancy, (3) other hormonal factors (such as studies on the menstrual cycle and menopause involving other organs or utilising haemodynamic or experimental modalities), (4) disease-related studies, (5) prematurity, (6) other paediatrics, (7) sex differences, (8) hormonal treatment (encompassing HRT and other hormonal treatments such as androgens or oestrogen receptor modulators like tamoxifen), and (9) other treatment-related studies. The studies to be included were divided into two groups: (1) studies focusing on the menstrual cycle and (2) studies comparing pre- and post-menopause in healthy adult women. In cases where articles could belong to more than one category, only the predominant category was selected.

### Data collection and risk of bias assessment

To offer a comprehensive overview of all incorporated sources, participant details, test characteristics, and key outcomes from the included studies were summarised in a standardised data collection template. We extracted general aspects such as title, authors, publication year, study design, journal and the country of affiliation of the main authors. Regarding the population, we collected data on sample size, ethnicity, age, and other specific characteristics. We registered imaging modalities, topographical region studied, methods of measurement extraction from the images, time of the day of the examination and laterality. Regarding the hormonal factors, we identified the main phase within the menstrual or post-menopausal stages as well as subphases, methods of phase measurement and confirmation, parity and other variables. We documented the main statistical tests used and whether adjustments were made. The Newcastle-Ottawa Score (NOS) was employed to evaluate the risk of bias in the included studies, assessing selection, comparability and exposure/outcome criteria [53]. The NOS scale for Case-Control Studies was adapted for cross-sectional studies, while the scale for Cohort Studies was adapted for longitudinal studies. Each article was assigned a specific score ranging from zero to nine stars, with a higher number of stars indicating a higher-quality study. The scoring scale can be found in Supplementary Material 3. Finally, if more than three studies provided quantitative measures of the same region and hormonal stage, a meta-analysis was conducted and visually presented as a forest plot. Standardised mean differences in the thickness of specific retinal areas between groups and 95% confidence intervals were obtained using a random-effects model. The heterogeneity of effect size estimates across studies was assessed using the *I*^2^ index. We utilised R version 4.3.0, released on the 21st of April 2023. Data were extracted from published articles accessible through online libraries affiliated with the authors’ organisations.

## Results

### Search and selection of data

**Fig. 1:**
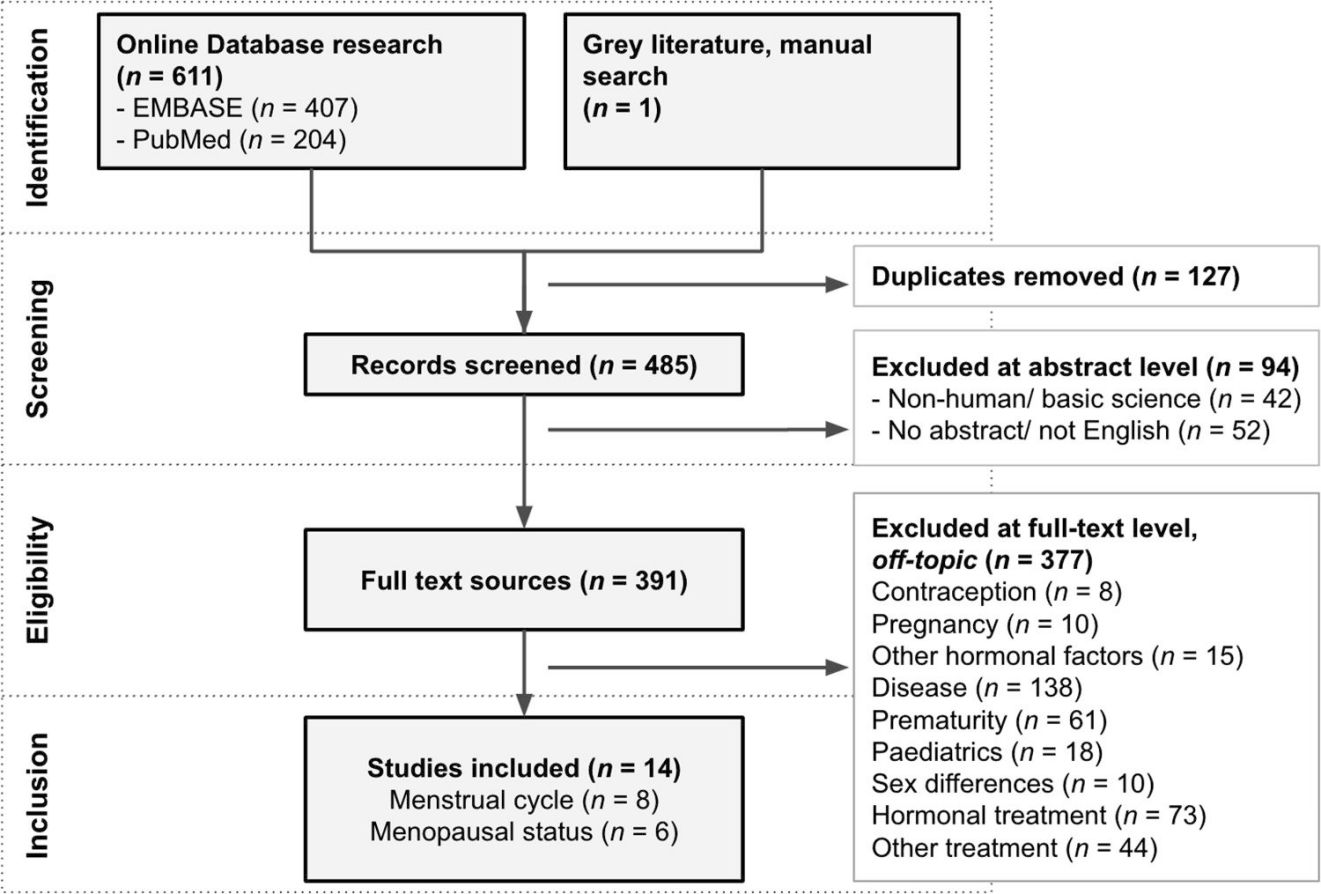
Study selection process in the format of a PRISMA-ScR flow diagram. PRISMA-ScR: Preferred reporting items for systematic reviews and meta-analyses extension for scoping reviews.

The full study selection process is presented as a PRISMA-ScR flow diagram (Fig. 1). We identified 612 references for analysis: 407 from Embase, 204 from PubMed, and 1 manually selected from grey literature. After removing duplicates, 485 underwent a first screening phase conducted in parallel by two reviewers (A.P.R.R. and E.I.) using a spreadsheet with metadata and abstracts. Disagreements were resolved through re-arbitration. The spreadsheet containing the 485 references and their subcategorisation as well as an accompanying README file are available online in our OSF project folder titled ‘Article selection’ (osf.io/bfwn3). A total of 391 items were retrieved in full and assessed for their compatibility with our inclusion/ exclusion criteria and a total of 14 studies met the criteria [54–67]. Among these, 13 [54–66] were original research articles published in peer-reviewed journals and 1 was an original research abstract from an international conference [67]. A total of 8 original studies [54–58, 64, 65, 67] examined menstrual cycle factors. Among them, a cross-sectional study explored relationships with cycle length and age of menarche [64] and 7 longitudinal studies investigated morphological changes across menstrual cycle phases [54–58, 65, 67]. The final 6 studies compared pre-menopausal and spontaneous post-menopausal women, in a cross-sectional design [59–63, 66]. A comprehensive overview of the key features of the incorporated studies can be found in Supplementary Material 4 and online in our OSF project folder titled ‘Included studies’ (osf.io/bfwn3).

### Study groups and ethnicity

In studies on menstrual cycle phases, Turkey led with the highest number of reports (4 articles) [54–56, 67], followed by Australia [64], the United States (US) [57], Japan [65], and China [58], each contributing one study. Only two studies provided information on participant ethnicity. The Australian study referenced ‘predominantly white ethnicity’ [64] while the US study provided demographic data on ethnicity. However, the small sample size (28 menstruating women) limited meaningful comparisons across ethnic groups [57]. Regarding studies on menopausal status, three studies were conducted in Turkey [59–61], two in Egypt [62, 63] and one in Nigeria [66]. None of the studies comparing pre- and post-menopause mentioned the ethnicity of the participants.

### Associations with the menstrual cycle

Table 1 provides a comprehensive overview of the main findings for the cited papers associating the menstrual cycle with macular, choroidal and disc structures.

**Table 1:** Summary of findings from studies investigating the association between menstrual cycle phases and macular, disc and choroidal structures.

Study	Phases of cycle	Imaging device	Sample size	Macula	Disc	Choroid
Lee S.S.Y. et al., [64]	NA	SD-OCT	494	NA	No relation of CDR or neuroretinal rim to age of menarche or cycle length.	NA
Akar et al., [54]	Follicular, ovulatory, late-luteal	SLO	38	NA	CDR increased and neuroretinal rim area decreased in luteal phase. No differences between follicular and ovulatory phases. RNFL: no statistical difference.	NA
Ulaş et al.,[55]	follicular, ovulatory, mid-luteal	SD-OCT, EDI	23	TRT, RNFL: no significant difference	Peripapillary RNFL: no significant difference	Subfoveal CHT decreased significantly in the mid-luteal phase
Ozcaliskan S. et al., [67]	follicular, ovulatory, mid-luteal	OCTA	21	SCP, DCP, FAZ: no significant difference	NA	NA
Guo et al., [58]	early-follicular, ovulatory, luteal	OCTA	62	SCP, FAZ: no significant difference. DCP Nasal and Inferior significantly thinner VD in the ovulatory phase	NA	NA
Fortepiani et al., [57]	first half, second half of the cycle	SD-OCT	28	Suboveal TRT: no statistical difference	NA	NA
Aşikgarip et al., [56]	follicular, ovulatory, mid-luteal	SD-OCT, EDI	36	NA	NA	Subfoveal CHT decreased significantly in the mid-luteal phase. CVI showed a significant change in mid-luteal phase
Kurahashi et al., [65]	late-follicular, mid-luteal	SD-OCT, EDI	15	NA	NA	Subfoveal CHT was significantly lower in the mid-luteal phase compared to the late follicular phase

The table includes information on the study, phases of the cycle included, imaging device used, sample size, and key findings for each applicable region.

#### Macula

Ulaş et al. conducted a study involving 23 healthy nulliparous females undergoing menstrual cycles, with a mean age of 26 ( ± 3 SD) years, using optical coherence tomography (OCT) at the follicular, ovulatory, and mid-luteal phases within one menstrual cycle. They found no statistical differences in total retinal thickness (TRT) or macular retinal nerve fibre layer (RNFL) [55]. Fortepiani et al. followed 28 women with a mean age of 26 years (±5 SD), of whom 16 were observed during both the first and second halves of a menstrual cycle, while the remaining 12 women were monitored over two or three cycles. They found no statistical differences in foveal thickness between the follicular and luteal phases on OCT [57]. In OCT angiography (OCTA) studies, Asri S.N. et al. observed no statistically significant differences in vessel density of the superficial capillary plexus (SCP), deep capillary plexus (DCP), and foveal avascular zone (FAZ) area between the follicular, ovulatory and mid-luteal phases [68]. On the other hand, Guo et al. conducted a study involving 62 women aged 27 (±2 SD) years, where they investigated the early-follicular, ovulatory and luteal phases controlling for ovulation through urinary luteinising hormone (LH) home-monitoring and adjusting the analysis to age, mean arterial pressure, spherical equivalent, axial length, and intraocular pressure. This study found no significant difference in vessel density of SCP or FAZ. However, they did observe that vascular density in the DCP within the nasal and inferior subfields was significantly lower during ovulation compared to the follicular or luteal phases (*p* < 0.001) [58].

#### Optic disc

In the aforementioned study, Ulaş et al. observed no statistically significant differences in peripapillary RNFL thickness on OCT within cycle phases among healthy young women [55]. Additionally, Akar et al. conducted a study involving 38 nulliparous, eumenorrheic women with a mean age of 26 (±4 SD) years, using serological hormonal confirmation of the cycle phase during follicular, ovulatory and late-luteal phases. They also found no significant difference in peripapillary RNFL thickness on OCT [54]. Concerning disc cupping, the latter group found an increased cup-to-disc ratio (CDR) and correspondingly decreased neuroretinal rim area during the late-luteal phase compared to the follicular and ovulatory phases (*p* < 0.001), with no significant differences observed between the follicular and ovulatory phases [54]. Finally, in a cross-sectional study, 10 females who had given birth at least once had smaller CDR (*p* < 0.001) and larger neuroretinal rim (*p* = 0.010) than nulliparous women, suggesting a protective effect of past pregnancies on CDR. However, no relation to the age of menarche or cycle length was found [64].

#### Choroid

**Fig. 2:**
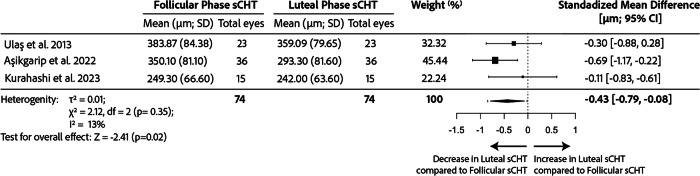
Meta-analysis comparing subfoveal choroidal thickness (sCHT) differences between follicular and luteal phases in longitudinal studies of healthy spontaneously menstruating women in the literature calculated through a Random effects Model. There was a thinner sCHT in the luteal phase compared to the follicular phase. CI confidence interval, SD standard deviation.

Three studies consistently found a significantly thinner subfoveal CHT during the luteal phase compared to follicular and ovulatory phases using OCT with enhanced depth imaging (EDI). A spreadsheet with the extracted quantitative data is available at our OSF page (osf.io/bfwn3) in the folder ‘Meta-Analysis’. A forest plot of the meta-analysis of these results can be found in Fig. 2. The random-effects model, based on the 3 studies [55, 56, 65], estimated a pooled effect size of −0.43 (95% CI: −0.79 to −0.08; *p* = 0.02), indicating a significant difference in subfoveal CHT between the two phases. The heterogeneity analysis showed low total heterogeneity (*I*^2^ = 12.53%), suggesting that ~12.53% of the total variability in effect sizes was due to true differences between studies rather than chance (*p* = 0.35). The study with the highest impact on the meta-analysis was Aşikgarip et al. which accounted for almost half of the participants, with a sample size of 36 [56] out of a total of 74 individuals. Additionally to the subfoveal region, they also measured a reduction in mid-luteal CHT in both the mean nasal and mean temporal quadrants [56]. In all three studies, healthy women were followed within a cycle and the time of examination was reported and remained consistent for each study. One study conducted examinations between 12 a.m. and 1 p.m. [55], another between 9 a.m. to 12 a.m. [56], and the last between 12 a.m. and 3 p.m., covering different times of the day.

### Associations with the menopausal status

Table 2 provides a comprehensive overview of the main findings of the cited papers associating menopausal status with macular, choroidal and disc structure.

**Table 2:** Overview of studies investigating the relationship between menopausal status and macular, disc and choroidal structures.

Study	Imaging device	Sample size, mean age (± SD)	Macula	Disc	Choroid
Ataş et al., [60]	SD-OCT, EDI	Pre-menop.: 72, 40 ( ± 8); Post-menop: 72, 57 ( ± 5)	Thinner post-menopausal TRT in all quadrants without adjustment. No difference, after age adjustment	Peripapillary RNFL: No significant difference, with or without age adjustment.	After adjusting for age, CHT remained significantly thinner in post-menopausal
Fathy et al., [62]	OCTA	Pre-menop: 50, 43 ( ± 2); Post-menop: 50, 55 ( ± 4)	NA	RNFL and peripapillary VD significantly thinner post-menopause. Oestrogen level was an independent predictor for whole image, peripapillary, superior and temporal VD.	NA
Alpogan & Tekcan, [59]	SD-OCT	Pre-menop: 47, 46 ( ± 3); Post-menop: 47, 53 ( ± 3)	Macular GCC: No significant difference, adjusting for age and BMI	Peripapillary RNFL: No significant difference, adjusting for age and BMI	NA
Çetinkaya Yaprak & Erkan Pota, [61]	SS-OCT, OCTA	Pre-menop: 45, 46; Post-menop: 40, 49	TRT, macular GCL and RNFL: no significant difference. SCP and DCP VDs: no statistical difference. Significant increase in both superficial and deep FAZ area in post-menopause. Choriocapillaris VD statistically lower in post-menopause	NA	CHT: no significant difference
Elghonemy et al., [63]	SD-OCT, EDI	Pre-menop: 25, 30 ( ± 3); Post-menop: 25, 54 ( ± 2)	TRT: no significant difference	Peripapillary RNFL: no significant difference	CHT thinner in post-menopause, no significance level presented.
Okonkwo O.N. et al., [66]	SD-OCT	pre-menopausal: 28, <45 post-menopausal: 56, >45	NA	NA	CHT thinner in post-menopause, not adjusted to age

The table includes the study reference, imaging device used, sample size with mean age (± standard deviation [SD]), and findings for the respective regions.

#### Macula

Four studies investigated macular TRT in both pre and post-menopausal women and did not find any statistically significant differences between the two groups [59–61, 63]. In the study by Ataş et al., which involved 72 pre-menopausal and 72 post-menopausal women with mean ages of 40 ( ± 8 SD) and 57 ( ± 5 SD) years respectively, a thinner TRT in all quadrants was observed in the post-menopausal group. However, this difference lost significance after age adjustments [60]. Similarly, Alpogan and Tekcan analysed 94 women, finding no statistically significant difference in macular ganglion cell complex (GCC) relating to menopausal status adjusting for age and BMI [59]. Finally, another study investigated macular ganglion cell layer and RNFL thickness in swept-source OCT in 45 pre-menopausal women and 40 post-menopausal women. The mean age of the two groups was very similar, with pre-menopausal women averaging 46 years and post-menopausal women 49 years. They found no statistically significant difference relating to menopause [61]. In the same study, OCTA revealed no statistically significant difference in vessel density (VD) for both SCP and DCP. However, it noted a significant increase in FAZ area in post-menopausal women (*p* = 0.013) in both SCP and DCP, as well as a reduction in choriocapillaris VD (*p* = 0.045) [61].

#### Optic disc

Peripapillary RNFL thickness between pre- and post-menopausal women was investigated in three studies and did not show significant differences in an unadjusted analysis [63], with or without age adjustment in another study [60] of after accounting for BMI [59]. Fathy et al. investigated 50 pre-menopausal and 50 post-menopausal women with peripapillary OCTA and found that peripapillary VD was significantly thinner in post-menopause (*p* < 0.001) with oestrogen level being an independent predictor for whole image, peripapillary, superior and temporal VD [62]. This study consistently investigated pre-menopausal women in the mid-follicular phase and measured oestrogen levels, in addition to questionnaire information.

#### Choroid

**Fig. 3:**
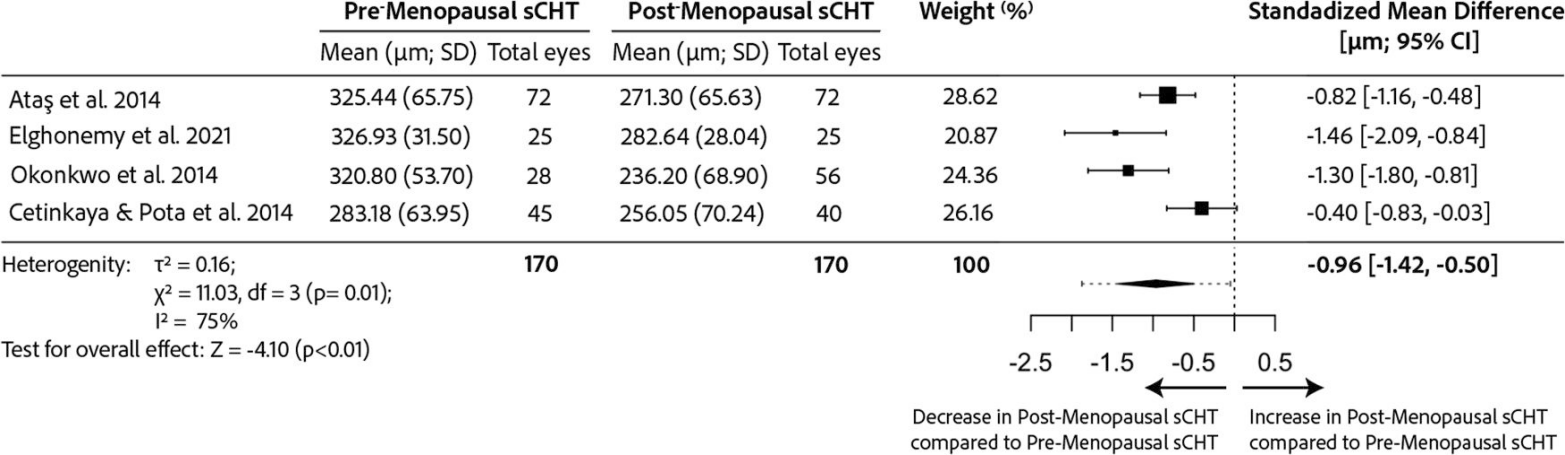
Meta-analysis comparing subfoveal choroidal thickness (sCHT) differences between pre- and postmenopausal healthy women in the literature calculated through a Random effects Model. There was a thinner sCHT in the post-menopausal group in unadjusted analysis. CI confidence interval, SD standard deviation.

Four studies compared the CHT of pre- and post-menopausal women [61, 63, 66, 69], as depicted in the meta-analysis shown in Fig. 3. Three of them reported a statistically significant reduction in the post-menopausal group, with significance persisting after age adjustment in one study (*p* = 0.005) [60]. On another hand, Cetinkaya & Pota found no difference in CHT between the two groups, even without age adjustment [61]. Neither of these studies referred to the time of the CHT measurement, not considering diurnal variation within individuals but all of them used an EDI system.

### Risk of bias analysis and general considerations

#### Menstrual cycle

Different methods were used to estimate menstrual cycle phases and the specific phases analysed also varied throughout the literature. While some studies compared women across two phases (follicular and luteal) [57, 65], others considered three phases by incorporating ovulation [54–56, 58, 67], with some specifying early, mid, or late subphases. We used the NOS criteria to assert the quality of nonrandomised studies and perform a risk of bias analysis of our included articles. The results are presented in Supplementary Material 5. According to the NOS, a high-quality study typically requires three or four stars in the selection domain, one star in the comparability domain, and two or three stars in the outcome domain. Among the six longitudinal studies assessing the menstrual cycle, only one study by Guo et al. [58] achieved a good quality score of 8 out of 9 stars. Its strength lay in the fact that it not only excluded contraception, HRT, and pregnancy but also utilised both self-report and hormonal confirmation of the cycle with at-home LH urinary tests. Furthermore, they accounted for confounding factors such as mean arterial pressure, spherical equivalent, axial length, and intraocular pressure in their analysis. Fortepiani et al. [57] and Akar et al. [54] were the next highest-scoring studies, each receiving 6 out of 9 stars. However, Fortepiani’s study relied solely on self-reported cycle phases, while Akar’s study did not make adjustments for confounders in their analysis. In general, all studies utilised self-report to calculate the cycle phase with only two studies confirming it with hormonal tests: one with temperature measurements and serum oestradiol, progesterone and luteinising hormone levels [54], and another measuring peak LH concentration in urine at home [58]. All studies considered only eumenorrheic women but exclusion criteria also varied. Two of them included only nulliparous individuals [54, 55], while five did not provide information on the number of previous pregnancies. Only two studies explicitly excluded lactating women [54, 56], four studies explicitly excluded participants using hormonal therapy and contraception [54–56, 58], while two studies permitted contraception and adjusted for it in their analyses [57, 64]. Included women were mostly in their twenties, with an average age ranging from 20 to 27 years old. All studies analysed ocular coherence-based metrics, and results for macular and optic disc parameters were automatically extracted using in-built software devices, except for CHT, which was manually measured. One study calculated the choroidal vascular index (CVI) by binarising EDI-OCT images with Image-J software [56].

#### Menopause

In the six menopausal studies, three used self-reporting to categorise participants [59, 61, 63], defining post-menopausal women as those who had experienced their last period spontaneously at least 12 months ago. Two studies employed serological tests, with one considering follicle-stimulating hormone (FSH) serum levels (>40 IU/L) [60] and the other serum oestradiol levels [62], while one study simply established an age cut-off at 45 years old [66]. Within the pre-menopausal participant groups, only one study explicitly identified the phase of the menstrual cycle, choosing the mid-follicular phase [62]. Five studies uniformly excluded HRT in their analyses [59–63], while only one study accounted for the influence of hormonal contraception [62]. The average age of post-menopausal women ranged from 48 to 60 years old. For the premenopausal group, the mean age ranged from 40 to 46 years old, nearing perimenopausal age, except for one study that included younger participants with a mean age of 30 (±3 SD) years old [63]. Similarly to menstrual cycle studies, OCT and OCTA results for macular and disc parameters were automatically extracted using the manufacturer’s built-in software, while choroidal thickness was manually measured in all studies. Two of these cross-sectional studies on menopause achieved good quality scores on the NOS scale [62, 69] and further results can be found in Supplementary Material 6. Both studies adjusted their analysis for age, but neither could achieve any significant results after this adjustment.

## Discussion

This review explores structural changes in the posterior pole during healthy women’s hormonal cycles using multimodal imaging. The posterior pole comprises multiple structures, and although our review did not restrict imaging modalities or regions, encompassing the macula, optic disc, and choroid, limited data and inconsistent reporting presented challenges in establishing meaningful associations from the included studies. Interestingly, studies in this review were mainly conducted by groups from Turkey and Egypt, with limited articles from countries where biomedical research funding and publications most commonly originate [70–72]. Understanding how hormonal cycles impact the posterior pole is crucial for addressing sex differences in pathology, such as those reported in vitreomacular disease or glaucoma, as previously highlighted. Globally, two out of three blind individuals are women [73]. While influenced to some extent by socioeconomic factors like healthcare accessibility and longer life expectancy, the lack of research on female-specific factors should not be ignored.

Throughout the menstrual cycle, the most consistently reported morphological change in the studies was observed in CHT. Subfoveal CHT was consistently thinner in the luteal phase compared to the follicular phase in all three studies. The authors hypothesised that this phenomenon might be attributed to vasoconstriction caused by increased sympathetic activity during the luteal phase when both oestradiol and progesterone are elevated. However, hemodynamic studies present conflicting evidence compared to these morphological findings. One of the most consistently observed effects of oestrogens and progesterone is an increase in vasodilator tone [74, 75], dependent on endothelial nitric oxide (NO) [76]. Oestradiol appears to enhance beta-adrenergic receptor-mediated vasodilation, counteracting alpha-adrenergic vasoconstriction [77]. This increase in sympathetic outflow was suggested to compensate for a greater NO release, maintaining balanced vascular resistance [78]. Karadeniz et al. conducted a study on 23 healthy women, examining retrobulbar circulation with serial colour Doppler ultrasonography during a normal menstrual cycle, and found that hemodynamic parameters remained stable throughout the menstrual cycle [79]. Another study by Haneda et al. proposed a decrease in choroidal blood flow velocity during the late follicular phase, indicating a potential vessel calibre increase in this phase [80]. The combined sample size from the three studies examined in our review, focusing on CHT totalled only 74 women. Although the time of measurement was standardised within each study, since there is no consensus, it does not always overlap between studies, introducing the potential confounding of diurnal CHT variation into this analysis. To enhance the generalisability of findings, more studies are necessary, with larger sample sizes and more robust research methodologies in future investigations.

Regarding macular and disc morphology, there were no significant changes across the menstrual cycle in any of the OCT layers analysed (TRT, GCC, and macular or peripapillary RNFL). This suggests a conserved structure despite potential fluctuations in blood flow and perfusion. There was not enough evidence to draw any trends on CDR, VD and FAZ on OCTA due to the limited number of studies and conflicting evidence. OCTA studies would be particularly promising in adding information to the circulation and autonomic regulation across the cycle.

Studies on pre- vs. post-menopausal changes were only cross-sectional, and as a group, they did not reveal any meaningful trends when adjusting their analysis for the effect of age or BMI. Since post-menopause is a later life stage, separating the effect of age from the hormonal decline is challenging. Their standardisation was poorer, without reference to the cycle phase of pre-menopausal women and without accounting for diurnal variations of choroidal thickness.

There are multiple limitations to this review. The small number of publications and the inconsistent methods used in the different studies to measure the cycle and menopause made it challenging to draw meaningful relationships. The inconsistent exclusion criteria made populations and research questions slightly different, even within the same topic. Most studies relied solely on questionnaire information to estimate hormonal levels, and the phases of the cycle analysed also differed. Many analyses were not adjusted for important confounders, such as age in menopausal studies, and there were no longitudinal studies on pre- and post-menopause or accounting for the hormonal irregularities typical of perimenopause. Longitudinal studies on the cycle never followed more than one cycle and did not consider intra-individual variability.

### Recommendations for future studies

To explore the effects of ovarian hormones, researchers routinely take advantage of life’s natural fluctuations in oestradiol and progesterone. To ensure the homogeneity of the study population when studying physiological variations within the menstrual cycle or menopause, it is important to exclude or account for women in different hormonal environments, such as those experiencing pregnancy, lactation, perimenopause, hormonal treatments, HRT, or contraception.

#### Determining menstrual cycle phase

Various methods exist for reporting menstrual cycle phases, each with its strengths and weaknesses. These include relying solely on self-reported information, utilising published ranges for ovarian hormone levels (with direct assays available in saliva, blood serum, blood plasma, and urine), combining self-report with hormonal levels or basal body temperature, and examining within-person changes in ovarian hormone levels collected multiple times over the cycle [50, 51, 81]. While self-reporting alone is very common, it is the weakest method in isolation. However, if the calculation of the menstrual cycle phase is transparent and standardised, using it as a proxy for hormonal action could be acceptable since it is the most cost-effective and minimally burdensome for participants [50, 51, 81]. Since it lacks the precision to identify specific cycle subphases, such as early, mid, or late phases, when used in isolation, the identification of ovulation exhibits the lowest accuracy and should not be considered [81]. Finally, it is recommended that cycle tracking by the participants be initiated a couple of months before answering self-report questionnaires and that ovarian hormonal levels are used in conjunction with self-reporting [50, 51, 81].

#### Determining menopausal status

Spontaneous menopause is defined as the permanent cessation of menstrual periods, established retrospectively after a woman has undergone 12 consecutive months of amenorrhoea without any apparent alternative cause [82]. Determining menopausal status is a more straightforward process, often defined by questionnaire. Longitudinal studies would be particularly valuable for understanding the impact of perimenopause through menopause within individuals, extending beyond chronological age. Caution is advised when including participants in perimenopause as controls since this period has irregular cycle lengths and anovulatory cycles. In the case of potential perimenopausal participants, the staging system ‘Stages of Reproductive Aging Workshop (STRAW + 10)’ for participants aged 40 and above helps in calculating their menopausal status [83]. Finally, in studies exploring pre- and post-menopausal changes, maintaining consistency in the menstrual cycle phase of the pre-menopausal group is crucial for obtaining reliable and comparable results.

#### Contraception

In the investigation of the natural menstrual cycle or menopause, it is recommended that cycling participants have experienced a minimum of two natural cycles after discontinuing hormonal contraceptives [81]. This approach allows for a more accurate representation of the physiological changes associated with the cycle. Furthermore, consistent exclusion of all types of hormonal contraception and not only oral contraceptives, including hormonal intra-uterine devices, implants, or injections, is essential to minimise confounding variables and enhance the internal validity of the research findings.

#### Other considerations

In CHT studies, it is crucial to account for the time of day, as diurnal changes can influence measurements and contribute to variations in outcomes [84]. Additionally, ethnicity should always be reported and accounted for due to normal variations within individuals from different groups [85]. Lastly, we encourage equally publishing negative results since this will contribute to a more comprehensive understanding of the field, avoiding publication bias and providing a well-rounded perspective that benefits all.

In summary, additional well-conducted, longitudinal cohort studies better describing female-specific variables are needed to evaluate the association between changes in posterior pole structures during the menstrual cycle and menopause. This could help in improving our understanding of the pathophysiology of posterior pole disorders in women.

## Supplementary information

Supplementary Material
