# Supplementary material for: Macular, choroidal and disc associations across women’s reproductive life stages: a scoping review from menarche to post-menopause
Source: Eye (Lond). 2025 Jan 15;39(3):402–11. doi: 10.1038/s41433-025-03592-w (PMC11794579; doi:10.1038/s41433-025-03592-w)
Supplement: Supplementary file 1 — Supplementary Material [file 41433_2025_3592_MOESM1_ESM.docx]

# Supplementary material

### 1. PRISMA-ScR Checklist

**Preferred Reporting Items for Systematic reviews and Meta-Analyses extension for Scoping Reviews (PRISMA-ScR) Checklist**

| **SECTION** | **ITEM** | **PRISMA-ScR CHECKLIST ITEM** | **REPORTED ON PAGE #** |
| --- | --- | --- | --- |
| **TITLE** | | | |
| Title | 1 | Identify the report as a scoping review. | 1 |
| **ABSTRACT** | | | |
| Structured summary | 2 | Provide a structured summary that includes (as applicable): background, objectives, eligibility criteria, sources of evidence, charting methods, results, and conclusions that relate to the review questions and objectives. | 1 |
| **INTRODUCTION** | | | |
| Rationale | 3 | Describe the rationale for the review in the context of what is already known. Explain why the review questions/objectives lend themselves to a scoping review approach. | 1-2 |
| Objectives | 4 | Provide an explicit statement of the questions and objectives being addressed with reference to their key elements (e.g., population or participants, concepts, and context) or other relevant key elements used to conceptualize the review questions and/or objectives. | 3 |
| **METHODS** | | | |
| Protocol and registration | 5 | Indicate whether a review protocol exists; state if and where it can be accessed (e.g., a Web address); and if available, provide registration information, including the registration number. | 3-4 |
| Eligibility criteria | 6 | Specify characteristics of the sources of evidence used as eligibility criteria (e.g., years considered, language, and publication status), and provide a rationale. | 4 |
| Information sources* | 7 | Describe all information sources in the search (e.g., databases with dates of coverage and contact with authors to identify additional sources), as well as the date the most recent search was executed. | 4 |
| Search | 8 | Present the full electronic search strategy for at least 1 database, including any limits used, such that it could be repeated. | Sup. Material 2 |
| Selection of sources of evidence† | 9 | State the process for selecting sources of evidence (i.e., screening and eligibility) included in the scoping review. | 4-5 |
| Data charting process‡ | 10 | Describe the methods of charting data from the included sources of evidence (e.g., calibrated forms or forms that have been tested by the team before their use, and whether data charting was done independently or in duplicate) and any processes for obtaining and confirming data from investigators. | 4-5 |
| Data items | 11 | List and define all variables for which data were sought and any assumptions and simplifications made. | 5 |
| Critical appraisal of individual sources of evidence§ | 12 | If done, provide a rationale for conducting a critical appraisal of included sources of evidence; describe the methods used and how this information was used in any data synthesis (if appropriate). | - |
| Synthesis of results | 13 | Describe the methods of handling and summarizing the data that were charted. | 6 |
| **RESULTS** | | | |
| Selection of sources of evidence | 14 | Give numbers of sources of evidence screened, assessed for eligibility, and included in the review, with reasons for exclusions at each stage, ideally using a flow diagram. | 6-7 |
| Characteristics of sources of evidence | 15 | For each source of evidence, present characteristics for which data were charted and provide the citations. | 6-7 |
| Critical appraisal within sources of evidence | 16 | If done, present data on critical appraisal of included sources of evidence (see item 12). | - |
| Results of individual sources of evidence | 17 | For each included source of evidence, present the relevant data that were charted that relate to the review questions and objectives. | 7-13 |
| Synthesis of results | 18 | Summarize and/or present the charting results as they relate to the review questions and objectives. | 7-13 |
| **DISCUSSION** | | | |
| Summary of evidence | 19 | Summarize the main results (including an overview of concepts, themes, and types of evidence available), link to the review questions and objectives, and consider the relevance to key groups. | 14-15 |
| Limitations | 20 | Discuss the limitations of the scoping review process. | 16 |
| Conclusions | 21 | Provide a general interpretation of the results with respect to the review questions and objectives, as well as potential implications and/or next steps. | 16-18 |
| **FUNDING** | | | |
| Funding | 22 | Describe sources of funding for the included sources of evidence, as well as sources of funding for the scoping review. Describe the role of the funders of the scoping review. | 18 |

JBI = Joanna Briggs Institute; PRISMA-ScR = Preferred Reporting Items for Systematic reviews and Meta-Analyses extension for Scoping Reviews.

* Where *sources of evidence* (see second footnote) are compiled from, such as bibliographic databases, social media platforms, and Web sites.

† A more inclusive/heterogeneous term used to account for the different types of evidence or data sources (e.g., quantitative and/or qualitative research, expert opinion, and policy documents) that may be eligible in a scoping review as opposed to only studies. This is not to be confused with *information sources* (see first footnote).

‡ The frameworks by Arksey and O’Malley (6) and Levac and colleagues (7) and the JBI guidance (4, 5) refer to the process of data extraction in a scoping review as data charting*.*

§ The process of systematically examining research evidence to assess its validity, results, and relevance before using it to inform a decision. This term is used for items 12 and 19 instead of "risk of bias" (which is more applicable to systematic reviews of interventions) to include and acknowledge the various sources of evidence that may be used in a scoping review (e.g., quantitative and/or qualitative research, expert opinion, and policy document).

*From:* Tricco AC, Lillie E, Zarin W, O'Brien KK, Colquhoun H, Levac D, et al. PRISMA Extension for Scoping Reviews (PRISMAScR): Checklist and Explanation. Ann Intern Med. 2018;169:467–473. [doi: 10.7326/M18-0850](http://annals.org/aim/fullarticle/2700389/prisma-extension-scoping-reviews-prisma-scr-checklist-explanation)

### 2. Search strategy and search string

| **Database** | **Search query** |
| --- | --- |
| **Pubmed**  **Medline**  **(All Fields)** | ("posterior pole" OR "retina*" OR "macula*" OR "fovea*" OR "choroid*" OR "optic nerve" OR "optic disc")  **AND**  ("optical coherence tomography" OR OCT OR "fundus photograph*" OR "confocal scanning laser ophthalmoscop*" OR angiography OR "near-infrared reflectance" OR "cup-to-disc" OR "cup to disc" OR vascular* OR density OR thickness)  **AND**  (menarche OR menstrua* OR menopaus* OR perimenopaus* OR premenopaus* OR pre-menopaus* OR postmenopaus* OR post-menopaus* OR climacteric OR "luteal phase" OR "follicular phase" OR ovulation) |
| **Embase**  **(All Fields + Text)** | Same as Pubmed |
| **Grey Literature Sources:**   - **Google / Google Scholar** - **Forward citation** | Free text |

### 3. Adapted Newcastle-Ottawa Scale (NOS)

| **NOS for Case Control Studies, adapted to  CROSS-SECTIONAL STUDIES** | **NOS for Cohort Studies, adapted to  LONGITUDINAL STUDIES** |
| --- | --- |
| **Selection (max: 4/4)** | |
| 1) Is the case definition adequate? | 1) Representativeness of the Cohort |
| a) yes, with independent validation ✵   *For instance, exclusion of hormonal confounders such as contraception, hormonal replacement therapy, pregnancy, hormonal medication and test, for instance pregnancy test.* | a) yes ✵     *Assessing the representativeness of exposed individuals in the community* |
| b) yes, eg record linkage or based on self reports | b) no description |
| c) no description | - |
| 2) Representativeness of the cases | 2) Ascertainment of Exposure |
| a) consecutive or obviously representative series of cases ✵ | a) yes, with independent validation ✵   *For instance, validation of pre- post menopausal definitions or menstrual cycle phases with self-report and hormonal confirmation, confirmation at multiple time points of the study regarding non-pregnancy, etc* |
| b) potential for selection biases or not stated | b) yes, eg record linkage or based on self reports |
| - | c) no description |
| 3) Selection of Controls | 3) Selection of individuals |
| a) community controls ✵ | a) community ✵ |
| b) hospital controls | b) hospital |
| c) no description | c) no description |
| 4) Is the definition of the control group adequate? | 4) Outcome of Interest Was Not Present at Start of Study |
| a) Description of source ✵   *For instance, validation of pre- post menopausal definitions or menstrual cycle phases with self-report and hormonal confirmation* | a) yes, with independent validation ✵   Confirmation of hormonal levels across the study and confirmation with repeated questionnaires/ self-report. |
| b) no description of source   *Or insufficient, such as using uniquely age cut-off* | b) yes, eg record linkage or based on self reports |
| - | c) no description |
| **Comparability (max 2/2)** | |
| 1) Comparability of cases and controls on the basis of the design or analysis | 1) Comparability of Cohort on the Basis of the Design or Analysis |
| a) Individuals must be matched in the design and/or confounders must be adjusted for in the analysis ✵ ✵   *For instance: control for age =* ✵*, control for other factor =* ✵ | a) Individuals must be matched in the design and/or confounders must be adjusted for in the analysis ✵ ✵   *For instance: control for age =* ✵*, control for other factor =* ✵ |
| **Exposure (max 3/3)** | **Outcome (max 3/3)** |
| 1) Ascertainment of exposure (max 1 point) | 1) Assessment of Outcome (max 1 point) |
| a) secure record, hormonal confirmation ✵ | a) Independent or blind assessment stated in the paper, or confirmation of the outcome by reference to secure records ✵ |
| b) structured interview where blind to case/control status ✵ | b) Record linkage ✵ |
| c) interview not blinded to case/control status | c) Self-report |
| d) written self report or medical record only | d) no description |
| e) no description | - |
| 2) Same method of ascertainment for cases and controls | 2) Was Follow-Up Long Enough? |
| a) yes ✵ | a) yes ✵ |
| b) no | b) no |
| 3) Non-Response rate | 3) Statistical test |
| a) same rate for both groups ✵ | a) The statistical test used to analyse the data is clearly described and appropriate ✵ |
| b) non respondents described | a) The statistical test is not appropriate, not described or incomplete |
| c) rate different and no designation | - |

### 4. Overview of the key features of the included studies

| **Title** | **Affiliation Country** | **Type of study** | **Reproductive Phase** | **Imaging device** | **Topograhical Region** |
| --- | --- | --- | --- | --- | --- |
| **(Lee S.S.Y. et al., 2019)** [**^64^**](https://paperpile.com/c/MKxJPl/9IyTx) | Australia | Cross Sectional | Menstrual | SD-OCT | disc |
| **(Akar et al., 2004)** [**^54^**](https://paperpile.com/c/MKxJPl/W9ebg) | Turkey | Longitudinal study | Menstrual | SLO | disc |
| **(Ulaş et al., 2013)** [**^55^**](https://paperpile.com/c/MKxJPl/2oZVB) | Turkey | Longitudinal study | Menstrual | SD-OCT, EDI | retina, choroid |
| **(Ozcaliskan S. et al., 2021)** [**^68^**](https://paperpile.com/c/MKxJPl/tMH1n) | Turkey | Longitudinal study | Menstrual | OCTA | retina |
| **(Guo et al., 2021)** [**^58^**](https://paperpile.com/c/MKxJPl/BV6G5) | China | Longitudinal study | Menstrual | OCTA | retina |
| **(Fortepiani et al., 2021)** [**^57^**](https://paperpile.com/c/MKxJPl/nfJ6f) | United States | Longitudinal study | Menstrual | SD-OCT | retina |
| **(Aşikgarip et al., 2022)** [**^56^**](https://paperpile.com/c/MKxJPl/qlEpD) | Turkey | Longitudinal study | Menstrual | SD-OCT, EDI | choroid |
| **(Kurahashi et al., 2023)** [**^65^**](https://paperpile.com/c/MKxJPl/S0oer) | Japan | Longitudinal study | Menstrual | SD-OCT, EDI | choroid |
| **(Ataş et al., 2014)** [**^60^**](https://paperpile.com/c/MKxJPl/zE7JD) | Turkey | Cross Sectional | Menopausal | SD-OCT, EDI | disc, retina, choroid |
| **(Fathy et al., 2022)** [**^62^**](https://paperpile.com/c/MKxJPl/RPT1r) | Egypt | Cross Sectional | Menopausal | OCTA | disc |
| **(Alpogan & Tekcan, 2022)** [**^59^**](https://paperpile.com/c/MKxJPl/Ev5E1) | Turkey | Cross Sectional | Menopausal | SD-OCT | disc, retina |
| **(Çetinkaya Yaprak & Erkan Pota, 2023)** [**^61^**](https://paperpile.com/c/MKxJPl/dkECd) | Turkey | Cross Sectional | Menopausal | SS-OCT, OCTA | retina, choroid |
| **(Elghonemy et al., 2022)** [**^63^**](https://paperpile.com/c/MKxJPl/22Lon) | Egypt | Cross Sectional | Menopausal | SD-OCT, EDI | retina, choroid, disc |
| **(Okonkwo O.N. et al., 2023)** [**^66^**](https://paperpile.com/c/MKxJPl/ADUg) | Nigeria | Cross Sectional | Menopausal | SD-OCT | choroid |

SD-OCT: Spectral Domain Optical Coherence Tomography; SS-OCT: Swept Source Optical Coherence Tomography; SLO: Scanning Laser Ophthalmoscope; EDI: Enhanced Depth Imaging; OCTA: Optical Coherence Tomography Angiography

### 5. Newcastle-Ottawa Scale (NOS) for Cohort Studies, adapted to LONGITUDINAL STUDIES

| **Study** | **Reproductive Phase** | **NOS Scale** | | | | | | | | |  |
| --- | --- | --- | --- | --- | --- | --- | --- | --- | --- | --- | --- |
|  |  | **Selection** | | | | **Comparability** | **Outcome** | | | **Overall** | **Quality** |
|  |  | **Representativeness** | **Ascerteinment of exposure** | **Participant Selection** | **Outcome not present at start** |  | **Assessment of Outcome** | **Adequate Follow-up length** | **Statistical test** |  |  |
| **(Akar et al., 2004)** | Menstrual | * | * | * | * | - | * | - | * | 6/9 | Poor |
| **(Ulaş et al., 2013)** | Menstrual | * | - | * | - | - | - | - | * | 3/9 | Poor |
| **(Ozcaliskan S. et al., 2021)** | Menstrual | * | - | * | - | - | - | - | - | 2/9 | Poor |
| **(Guo et al., 2021)** | Menstrual | * | * | * | * | * * | * | - | * | 8/9 | Good |
| **(Fortepiani et al., 2021)** | Menstrual | * | - | * | - | * * | - | * | * | 6/9 | Poor |
| **(Aşikgarip et al., 2022)** | Menstrual | * | - | * | - | - | - | - | * | 3/9 | Poor |
| **(Kurahashi et al., 2023)** | Menstrual | * | - | * | - | - | - | - | * | 3/9 | Poor |

### 6. Newcastle-Ottawa Scale (NOS) for Case-Control Studies, adapted to CROSS-SECTIONAL STUDIES

| **Study** | **Reproductive Phase** | **NOS Scale** | | | | | | | | |  |
| --- | --- | --- | --- | --- | --- | --- | --- | --- | --- | --- | --- |
|  |  | **Selection** | | | | **Comparability** | **Exposure** | | | **Overall** | **Quality** |
|  |  | **Case Definition** | **Case Representativeness** | **Control Selection** | **Control Definition** |  | **Ascertainment of exposure** | **Same Ascertainment Case and Control** | **Non-response Rate** |  |  |
| **(Lee S.S.Y. et al., 2019)** | Menstrual | * | * | * | * | * * | - | * | * | 8/9 | Good |
| **(Ataş et al., 2014)** | Menopause | * | * | * | * | * | * | * | * | 8/9 | Good |
| **(Fathy et al., 2022)** | Menopause | * | * | * | * | * * | * | * | * | 9/9 | Good |
| **(Alpogan & Tekcan, 2022)** | Menopause | - | - | * | - | * * | - | * | * | 5/9 | Poor |
| **(Çetinkaya Yaprak & Erkan Pota, 2023)** | Menopause | - | - | * | - | - | - | * | * | 3/9 | Poor |
| **(Elghonemy et al., 2022)** | Menopause | - | - | * | - | - | - | * | * | 3/9 | Poor |
| **(Okonkwo O.N. et al., 2023)** | Menopause | - | - | * | - | - | - | * | - | 2/9 | Poor |
|  | Case and Control groups can be seen as groups 1 and 2, such as pre-menopausal and post-menopausal or nulliparous and multiparous groups | | | | | | | | | |  |
